# A new carpenter ant, *Camponotus parabarbatus* (Hymenoptera: Formicidae) from India

**DOI:** 10.3897/BDJ.2.e996

**Published:** 2014-02-18

**Authors:** Himender Bharti, Aijaz Ahmad Wachkoo

**Affiliations:** † Punjabi University, Patiala, India

**Keywords:** India, Himalaya, Formicinae, *
Camponotus
*, new species

## Abstract

A new species of carpenter ant, collected in the Shivalik range of Himalaya is described and illustrated based on the worker and gyne castes under the name *Camponotus parabarbatus* sp. n. Presence of dense, short setae on gena and ventral surface of head resembles it most to *Camponotus barbatus* Roger, 1863 distributed in Southeast Asia. A regional identification key of *Camponotus* species is provided from the Shivalik hills of Indian Himalaya.

## Introduction

Carpenter ants are among the largest and most common ants in the world and are found in all biogeographical regions (Bolton 2012, Bolton et al. 2007). Currently 1,058 species, 495 subspecies and 31 fossil species of *Camponotus* are known worldwide (Bolton 2012), with 62 species and subspecies reported from India (Bharti 2011). Despite their large size and abundance, carpenter ants are difficult to identify. Although Karmaly and Narendran (2006) compiled the *Camponotus* of India, their taxonomy in India remains problematic and chaotic. Taxonomy of *Camponotus* in India needs attention as Bharti (2011) highlights major changes in the taxa included earlier by Karmaly and Narendran (2006).

The significant contributions pertinent to this study include Bingham (1903), Menozzi (1939), Morisita et al. (1991), Wu and Wang (1995), Radchenko (1996), Zhou (2001), Karmaly and Narendran (2006) and Terayama (2009). Here we describe a new Indian species, *Camponotus parabarbatus* sp. n., collected in Shivalik range of Northwest Himalaya. An identification key of *Camponotus* species is provided from the Shivalik hills of Indian Himalaya.

## Materials and methods

The specimens were collected by hand in the foothills of Indian Himalaya, the Shivalik range. Specimen examination was conducted with a Nikon SMZ 1500 stereomicroscope. For digital images, an MP evolution digital camera was used on the same microscope using Auto-Montage (Syncroscopy, Division of Synoptics, Ltd.) software. These images were processed with Adobe Photoshop CS5. Holotype and paratypes have been deposited in PUPAC, Punjabi University Patiala Ant Collection, Patiala. One paratype will be deposited at BMNH, Natural History Museum, London, UK and one at California Academy of Sciences, San Francisco, USA. Morphological definitions for measurements (accurate to 0.01 mm) include:

TL (Total Length) – HL+ML+PL+GL;

HL (Head Length) – length of head, excluding mandibles, measured in a straight line from anteriormost point of median clypeal margin to midpoint of occipital margin in full-face view;

HW (Head Width) – maximum width of head, measured in full-face view;

EL (Eye Length) – maximum length of eye as measured in oblique view of the head to show full surface of eye;

SL (Scape Length) – straight-line length of antennal scape excluding condylar bulb;

ML (Mesosoma Length) – diagonal length of mesosoma in lateral view from the point at which pronotum meets cervical shield to posteroventral corner of mesosoma;

PW (Pronotum Width) – maximum width of pronotum in dorsal view;

PL (Petiole Length) – In profile, the maximum length of the petiole node, measured in a straight horizontal line from immediately above the dorsal base of the anterior petiolar tubercle to the posterior margin;

mTbL (midtibia Length) – maximum length of midtibia in lateral view, with tibial base and apex in the same plane of focus, and with tibia at right angle to femur;

hTbL (hindtibia Length) – maximum length of hindtibia in lateral view, with tibial base and apex in same plane of focus, and with tibia at right angle to femur;

GL – length of the gaster in lateral view from the anteriormost point of first gastral segment to the posteriormost point of the last segment.

## Taxon treatments

### Camponotus parabarbatus

Bharti & Wachkoo, 2014 sp. n.

urn:lsid:zoobank.org:act:2A5E348D-3C27-4194-B147-9CACF67854CF

#### Materials

- **Type status:** Holotype. **Occurrence:** recordedBy: Aijaz A. Wachkoo; individualCount: 1; sex: worker; **Location:** country: India; stateProvince: Himachal Pradesh; locality: Rewalsar; verbatimElevation: 1360 m; verbatimLatitude: 31.6345°N; verbatimLongitude: 76.8343°E
- **Type status:** Paratype. **Occurrence:** recordedBy: Aijaz A. Wachkoo; individualCount: 5; sex: 2 workers, 3 gynes; **Location:** continent: Asia; country: India; stateProvince: Himachal Pradesh; locality: Rewalsar; verbatimElevation: 1360 m; verbatimLatitude: 31.6345°N; verbatimLongitude: 76.8343°E
- **Type status:** Paratype. **Occurrence:** recordedBy: Aijaz A. Wachkoo; individualCount: 6; sex: workers; **Location:** continent: Asia; country: India; stateProvince: Uttarakhand; verbatimElevation: 640 m; verbatimLatitude: 30.3416°N; verbatimLongitude: 77.9903°E; **Record Level:** institutionCode: Forest Research Institute
- **Type status:** Paratype. **Occurrence:** recordedBy: Aijaz A. Wachkoo; individualCount: 8; sex: workers; **Location:** continent: Asia; country: India; stateProvince: Uttarakhand; locality: Rajaji Forest Area; verbatimElevation: 660 m; verbatimLatitude: 30.2483°N; verbatimLongitude: 77.9878°E

#### Description

**Description of worker** (Fig. 1):

**Worker measurements:** TL: 5.10–6.85, HL: 1.23–1.98, HW: 0.92–1.70, EL: 0.32–0.41, SL: 1.15–1.39, ML: 1.87–2.38, PW: 0.77–1.15, PL: 0.19–0.23, mTbL: 1.00–1.06, hTbL: 1.36–1.44, GL 1.80-2.28 (n = 11).

Head: Head subtriangular, longer than wide in major worker (HW/HL = 0.86, n = 1), with arched margins laterally, posterior margin shallowly concave (Fig. 1a), distinctly elongate in minor worker (HW/HL = 0.75–0.77, n = 10), subrectangular with subparallel lateral margins and convex posterior margin; frontal carinae sinuous; clypeus in full-face view with anterior margin projected beyond anterior margin of gena; anterolateral corner of clypeus forming right angle, carinate in major worker, in minor worker clypeus relatively less carinate, with anterior margin only slightly extending beyond anterior margin of gena, anterolateral corner broadly rounded; scape short (SL/HW = 0.68), fails to reach occipital margin in major worker, distinctly elongate in minor worker (SL/HW = 1.18–1.44) surpassing posterior margin by about 0.33 of its length; mandible with six teeth in minor and seven in major with seventh tooth reduced.

Mesosoma: Mesosomal outline in lateral view smoothly arched; propodeal dorsum forming obtuse angle with declivity (Fig. 1c); propodeum compressed laterally; propodeal spiracle round; tibia tubular.

Petiole: petiolar scale broad, dorsally convex.

Sculpture: Head microreticulate, reticulation coarser on gena; mesosoma finely reticulate, gastral reticulations even feebler, appearing gently transversally striate. Mandible and scape with scattered punctures. Entire body shiny.

Vestiture: Pilosity yellowish; head, mesosoma, and all gaster segments with dense, erect, long setae; gena, entire ventral surface of head and mandible with dense shorter erect and suberect setae; scape with short, subapressed hairs; hindtibia without row of spiny bristles on ventral margin in addition to 3–4 suberect setae at distal end near spurs; body covered with very short, appressed, white pubescence, more distinct on head and gaster.

Color: Body black, regardless of size: antenna and leg reddish brown; trochanters yellow brown.

**Description of Gyne** (Fig. 2):

**Gyne measurements:** TL: 9.06–9.25, HL: 1.97–2.00, HW: 1.48–1.55, EL: 0.51–0.56, SL: 1.26–1.27, ML: 2.74–2.94, PL: 0.23–0.28, mTbL: 1.12–1.14, hTbL: 1.53–1.54; GL 4.03-4.12 (n = 3).

As in major worker, with modifications expected for caste and the following differences: head more elongate, sides relatively straight, occipital margin convex; mandible, clypeus and gena brownish. Head narrower than in conspecific major workers; mandible 7 toothed; scape barely reaches the posterior margin of head. Reticulate sculpture more pronounced on head; scutum with scattered wide, shallow punctures. Propodeum dorsum forms right angle with declivity.

#### Etymology

The species epithet *parabarbatus* is a compound word meaning “similar to *barbatus*”.

#### Distribution

This species seems to be rare in the Shivalik range of Northwest Himalaya although collected from both forested and non-forested areas of the region. Most workers were collected from vegetation while gynes and some workers were found under a large stone.

#### Notes

*Camponotus parabarbatus* resembles to the *Camponotus barbatus* distributed in Southeast Asia (Bolton et al. 2007) but can be easily distinguished from the latter. The head of the major worker of *Camponotus parabarbatus* is subtriangular with a shallowly concave posterior margin, the eyes well within the lateral cephalic margins, and the scape barely touches the posterior margin of head, while in *Camponotus barbatus* majors the head is subrectangular with gently convex posterior margin, eyes almost touching the lateral cephalic margins, scape surpasses the posterior margin of head by about 0.25 of its length. In *Camponotus parabarbatus* minor workers, the scape surpasses the posterior margin of head by about 0.33 of its length, whilst in *Camponotus barbatus* the scape does so by half its length. Additionally, *Camponotus parabarbatus* is uniformly jet-black whereas *Camponotus barbatus* is red brown in color.

## Identification Keys

### Key to the workers of *Camponotus* in Shivalik hills of Indian Himalaya

**Table:** 

1	Mesosomal profile continuous, forming a regular arch; the metanotal groove very shallow	5
–	Mesosomal profile discontinuous, not forming a regular arch; interrupted at the deep metanotal groove	2
2	Propodeal spiracle round or oval	3
–	Propodeal spiracle elongate, slit shaped	4
3	Pronotum dentate; body very densely pilose; hindtibia without spiny bristles on ventral margin	***Camponotus wasmanni* Emery**
–	Pronotum edentate; body sparsely pilose; hindtibia with spiny bristles on ventral margin	***Camponotus opaciventris* Mayr**
4	Petiole emarginate above; entirely black	***Camponotus horseshoetus* Datta & Raychaudhuri**
–	Petiole rounded above; head and mesosoma reddish, gaster blackish	***Camponotus nirvanae* Forel**
5	Gaster covered with fine sericeous pubescence	***Camponotus parius* Emery**
–	Gaster without any fine sericeous pubescence	6
6	Clypeus in full-face view with broadly rounded anterolateral corner; free margin distinctly emarginated medially	***Camponotus himalayanus* Forel**
–	Clypeus in full-face view with right-angled anterolateral corner; free margin entire	7
7	Tibiae compressed, prismatic	9
–	Tibiae tubular, not prismatic	8
8	Black and shining; setae on lateral and ventral sufraces of head dense	***Camponotus parabarbatus* sp. n.**
–	Mesosoma light brown, head and gaster blackish, relatively dull; setae on lateral and ventral surfaces of head sparse	***Camponotus oblongus binominatus* Forel**
9	Hind tibiae without longitudinal row of spiny bristles on ventral margin in addition to 3–4 suberect setae apically, near spurs	10
–	Hindtibia with at least one row of spiny bristles on ventral margin	11
10	Head and gaster blackish-brown or black; mesosoma and leg yellow-brown to ferruginous-red	***Camponotus mitis* (Smith, F.)**
–	Body uniformly dark black	***Camponotus lamarckii* Forel**
11	Body completely black	***Camponotus compressus* (Fabricius)**
–	Body not completely black	12
12	Head, mesosoma and leg ferruginous-red to reddish-brown; gaster reddish brown or blackish-brown	***Camponotus sylvaticus basalis* Smith, F.**
–	Head black; mesosoma and gaster and legs partly yellow brown	***Camponotus kattensis* Bingham**

## Supplementary Material

XML Treatment for Camponotus parabarbatus

## Figures and Tables

**Figure 1a.**
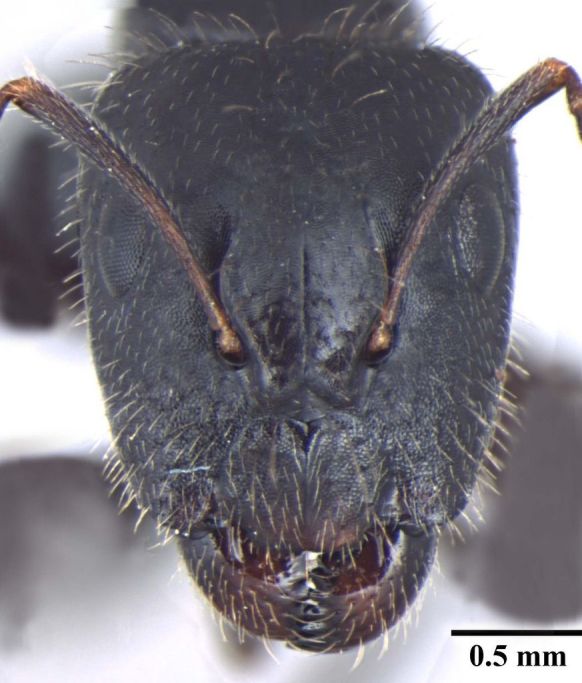
Major worker Head, full face view.

**Figure 1b.**
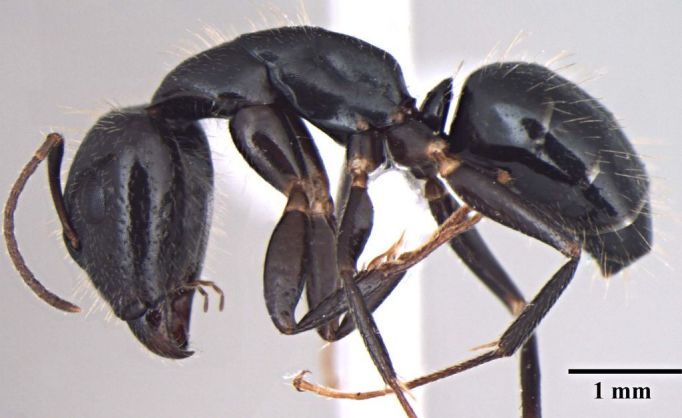
Major worker Body, lateral view.

**Figure 1c.**
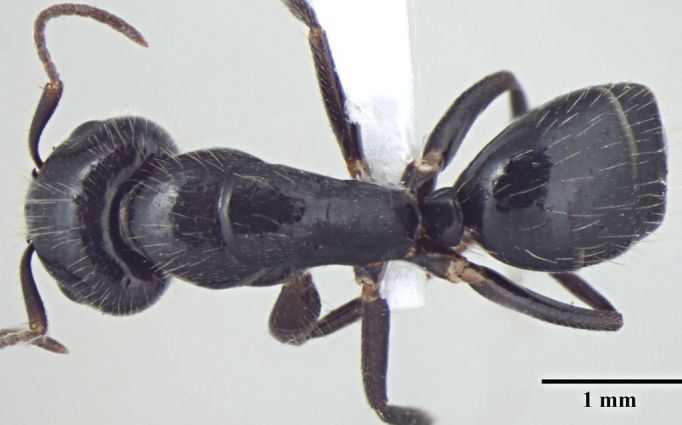
Major worker Body, dorsal view.

**Figure 1d.**
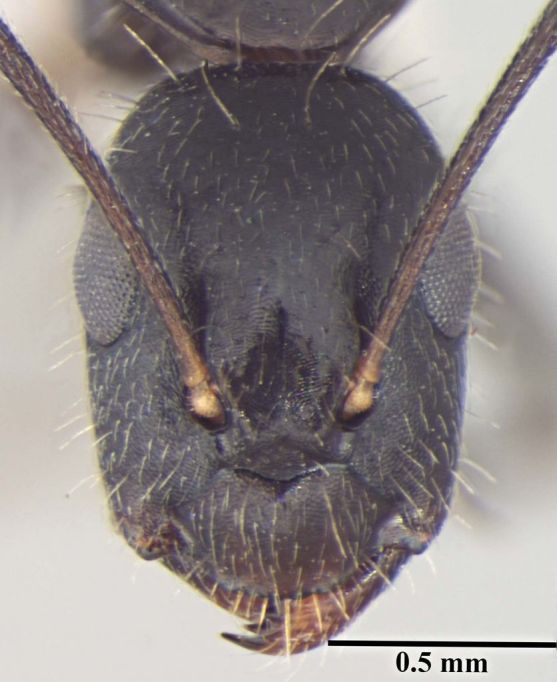
Minor worker Head, full face view.

**Figure 1e.**
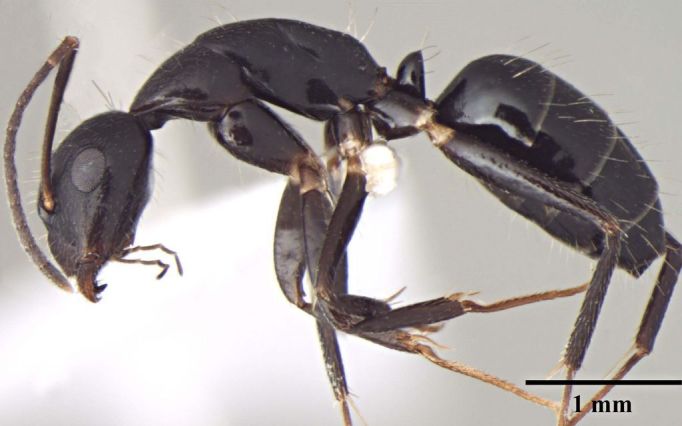
Minor worker Body, lateral view.

**Figure 1f.**
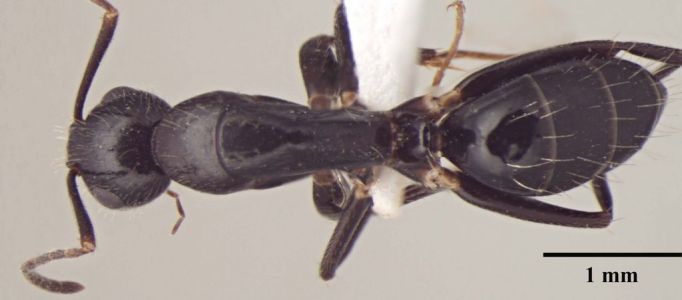
Minor worker Body, dorsal view.

**Figure 2a.**
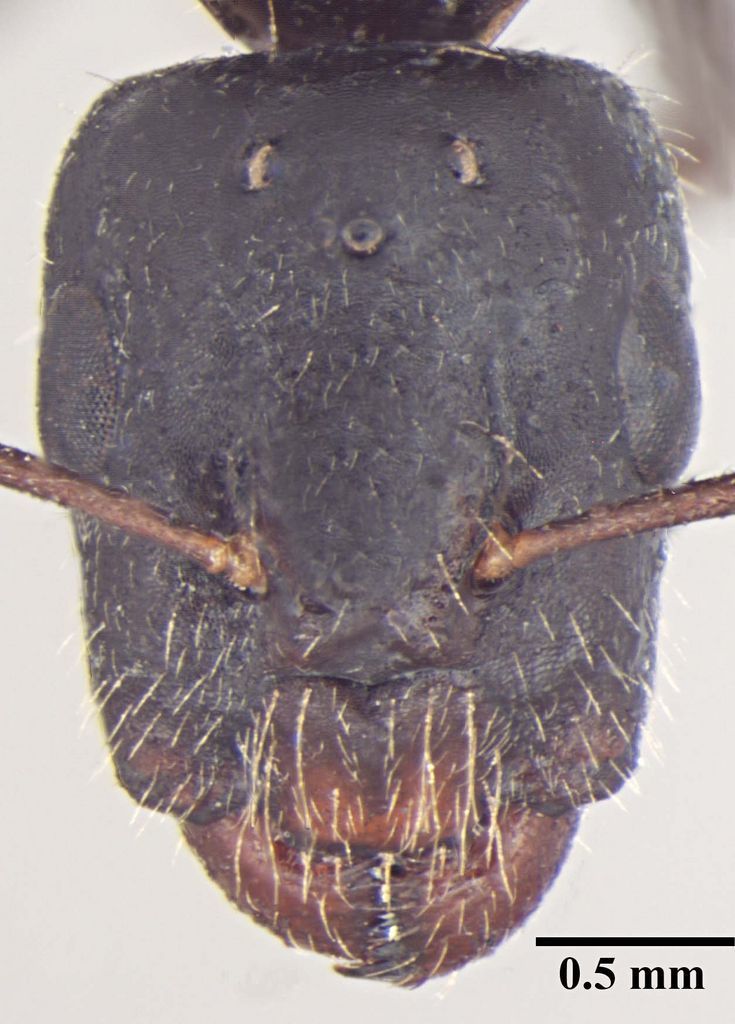
Gyne Head, full face view.

**Figure 2b.**
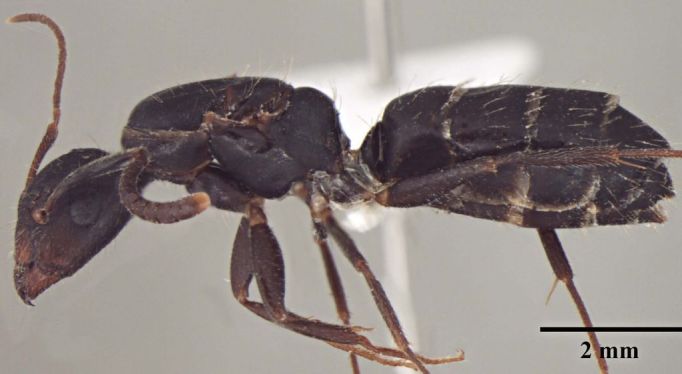
Gyne Body, lateral view.

**Figure 2c.**
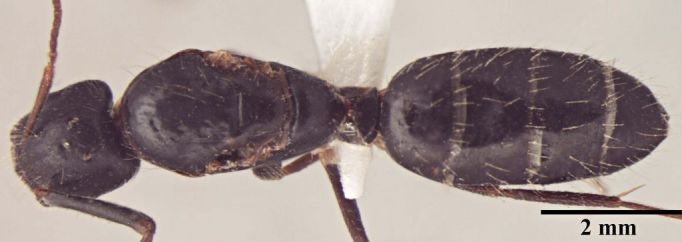
Gyne Body, dorsal view.
